# Band Gap of Pb(Fe_0.5_Nb_0.5_)O_3_ Thin Films Prepared by Pulsed Laser Deposition

**DOI:** 10.3390/ma14226841

**Published:** 2021-11-12

**Authors:** Nicole Bartek, Vladimir V. Shvartsman, Houssny Bouyanfif, Alexander Schmitz, Gerd Bacher, Selina Olthof, Svetlana Sirotinskaya, Niels Benson, Doru C. Lupascu

**Affiliations:** 1 Institute for Materials Science, Center for Nanointegration Duisburg-Essen (CENIDE), University of Duisburg-Essen, 45141 Essen, Germany; vladimir.shvartsman@uni-due.de (V.V.S.); doru.lupascu@uni-due.de (D.C.L.); 2 Laboratoire de Physique de la Matière Condensée UR2081, Université de Picardie Jules Verne, 80039 Amiens, France; houssny.bouyanfif@u-picardie.fr; 3 Electronic Materials and Nanostructures and Center for Nanointegration Duisburg-Essen (CENIDE), University of Duisburg-Essen, 47057 Duisburg, Germany; alexander.schmitz@uni-due.de (A.S.); gerd.bacher@uni-due.de (G.B.); 4 Institute for Physical Chemistry, University of Cologne, 50939 Cologne, Germany; solthof@uni-koeln.de; 5 Institute of Technology for Nanostructures, Center for Nanointegration Duisburg-Essen (CENIDE), University of Duisburg-Essen, 47057 Duisburg, Germany; svetlana.sirotinskaya@uni-due.de (S.S.); niels.benson@uni-due.de (N.B.)

**Keywords:** lead iron niobate, thin films, pulsed laser deposition, band gap, optical properties, photovoltaics

## Abstract

Ferroelectric materials have gained high interest for photovoltaic applications due to their open-circuit voltage not being limited to the band gap of the material. In the past, different lead-based ferroelectric perovskite thin films such as Pb(Zr,Ti)O_3_ (Pb,La)(Zr,Ti)O_3_ and PbTiO_3_ were investigated with respect to their photovoltaic efficiency. Nevertheless, due to their high band gaps they only absorb photons in the UV spectral range. The well-known ferroelectric PbFe_0.5_Nb_0.5_O_3_ (PFN), which is in a structure similar to the other three, has not been considered as a possible candidate until now. We found that the band gap of PFN is around 2.75 eV and that the conductivity can be increased from 23 S/µm to 35 S/µm during illumination. The relatively low band gap value makes PFN a promising candidate as an absorber material.

## 1. Introduction

The photovoltaic effect, which allows for the conversion of light to electricity, is considered one of the most promising directions of “green” energetics. The field of photovoltaic materials is dominated by devices based on doped silicon (Si) involving a p-n junction. The exciton binding energy in Si is smaller than the thermal activation energy, which makes the charge carrier generation easy. Due to an electric field arising within a space charge region formed around the p–n junction, the separation of photo-generated electrons and holes occurs. However, the manufacturing of silicon solar cells is quite demanding, making the production expensive. In order to overcome this problem, other highly efficient materials, e.g., hybrid halide perovskites, which can potentially be deposited on large areas using printing processes, are being researched [1,2]. Nevertheless, these materials are not long-lasting due to their degradation through humidity, temperature, and UV light [3]. Other promising materials could be ferroelectric perovskites due to their bulk photovoltaic effect [4]. The bulk photovoltaic effect has been known since 1966 [5]. It occurs in non-centrosymmetric crystals and depends on the ferroelectric polarization. When a poled ferroelectric is illuminated, the photons are absorbed and charge carriers are generated in the material. Due to the polarization-induced depolarization electric field, the electrons and holes are driven to the opposite electrodes [6]. Moreover, the value of the open-circuit voltage in ferroelectrics is not limited by the band gap of the material but can be several times higher [6,7,8]. Nevertheless, the photocurrent in bulk materials is rather small due to the scattering of photoinduced charge carriers on defect sites but can be increased with decreasing layer thickness. This makes ferroelectrics—especially in the form of thin films—an interesting candidate for photovoltaic applications [6]. Many studies were conducted on lead-based ferroelectric perovskites such as, e.g., Pb(Zr,Ti)O_3_ (PZT) [9,10,11,12,13], (Pb,La)(Zr,TiO)_3_ (PLZT) [6,7], and PbTiO_3_ (PTO) [14]. However, the absorbance of photons and thus also the photovoltaic efficiency is strongly dependent on the band gap. For these materials it is approximately 3.6 eV for PZT [11,12,15] and PTO [16,17,18] and larger than 3.35 eV for PLZT [19,20]. These band gaps limit the absorption of these materials to the ultraviolet spectrum, which makes up only around 8% of the solar spectrum [16]. Therefore, these materials only reach an efficiency of 0.75% for PZT [13,21], ~0.28% for PLZT [6], and 0.05% for PTO [14]. Based on their high absorption in the UV spectral range, these materials could, however, still be used, e.g., in tandem solar cells. Recently, multiferroic BiFeO_3_ (BFO) has attracted significant attention due to a relatively narrow band gap of 2.2–2.7 eV, promising a significant improvement in photovoltaic performance [22,23] compared to the aforementioned materials. Interestingly, another well-known multiferroic, namely lead iron niobate (PbFe_0.5_Nb_0.5_O_3_, PFN), has remained out of sight of the photovoltaic community. Ferroelectric, magnetic, and magnetoelectric properties of PFN were studied in detail [24,25,26,27]. The material has a ferroelectric–paraelectric phase transition in the range of 370–390 K [28] and undergoes a transition into the antiferromagnetic state at 143 K [29]. However, little is known about its optical properties and only few publications adressed the band gap on this material [30,31].

In this article, we report on a study of the optical properties of epitaxial PFN thin films produced by pulsed laser deposition. Several experimental methods were applied to evaluate the band gap of the highly homogeneous 100 nm thick PFN films. The data show that PFN is a promising candidate to be used for photovoltaic devices.

## 2. Material and Methods

**Figure 1:**
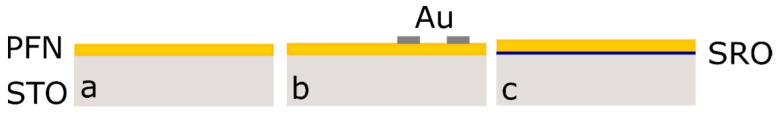
Different sample structures for the different measurements: (**a**) for XRD, RHEED, and UV-Vis; (**b**) for TLM; (**c**) for IPES/UPS.

PFN films were grown on commercial (001)-oriented SrTiO_3_ or SrTiO_3_/SrRuO_3_ (the thickness of the SrRuO_3_ layer was 50 nm) substrates (PLD Targets, London, UK) by pulsed laser deposition (PLD) with a KrF excimer laser (λ = 248 nm; see Figure 1a,c). The deposition parameters were as follows: a substrate temperature of 700 °C, an oxygen pressure of 0.2 mbar, a distance from the target to the substrate of 5.5 cm, and a laser fluence of around 2 J/cm^2^ (laser energy 60 mJ, laser spot area 3 mm^2^). The number of laser pulses was 5000 at a frequency of 3 Hz. A ceramic target for deposition was synthesized using the solid-state method: stoichiometric amounts of PbO (99.99%, Alfa Aesar, Kandel, Germany) with 10 wt. % lead excess, pre-milled Fe_2_O_3_ (99.9%, Alfa Aesar, Kandel, Germany), and Nb_2_O_5_ (99.9%, Alfa Aesar, Kandel, Germany) were mixed by a planetary ball mill for 4 h. The obtained mixture was calcinated at 850 °C for 2 h in oxygen atmosphere. The calcinated powder was ground, pressed in pellets, and also sintered at 1050 °C for 2 h in an oxygen atmosphere.

An Empyrean diffractometer (Malvern PANalytical, Kassel, Germany) with a CuKα_1_ source was used for verifying the phase content of the films in the 2*θ*-range of 15–80° with a 0.013° step size. The epitaxial film growth was checked with the help of pole figures measured in the same 2*θ*-range with a tilting angle *ψ* from 40 to 50° and a rotating angle from 0° to 360° in 1° increments, respectively. A high-resolution D8 Bruker (CuKα_1_ monochromator source λ = 1.54056 Å) four circles diffractometer was used for examining the crystallinity of the films by the analysis of rocking curves and the layer thickness with reflectivity measurements.

In situ, Reflection High Energy Electron Diffraction (RHEED) measurements were performed to check the surface quality and in-plane epitaxial relationship.

In order to determine the band gap, two different techniques were used. On the one hand, transmission spectra were recorded with a Shimadzu UV2550 (Shimadzu Deutschland GmbH, Duisburg, Germany) double-beam spectrometer in the range from 300 nm to 850 nm using an integrating sphere in transmission geometry to reduce scattering. On the other hand, direct and inverse photoelectron spectroscopy (UPS and IPES) were employed to analyze the occupied and unoccupied density of states, respectively. The measurement setup is described in detail in ref. [32].

Additionally, transmission line method (TLM) measurements were performed to detect electrical responses upon illumination of the sample. Therefore, Au electrodes were evaporated horizontally onto the film with a distance of 800 µm, as shown in Figure 1b. A two-point contact method was used instead of a 4-point contact, as it is assumed that the sheet resistance of the sample (GΩ range) is greater than the contact resistance between the Au electrode and the PFN. The voltage was swept between -10 V and +10 V in the dark and under illumination with a white light source.

## 3. Results and Discussion

**Figure 2:**
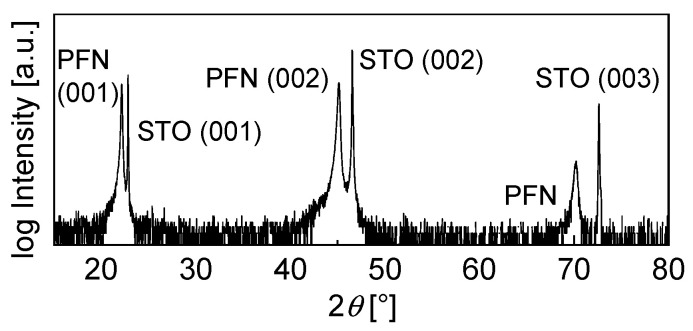
*θ*-2*θ* XRD spectra of the PFN film deposited on an STO (001) substrate via PLD.

**Figure 3:**
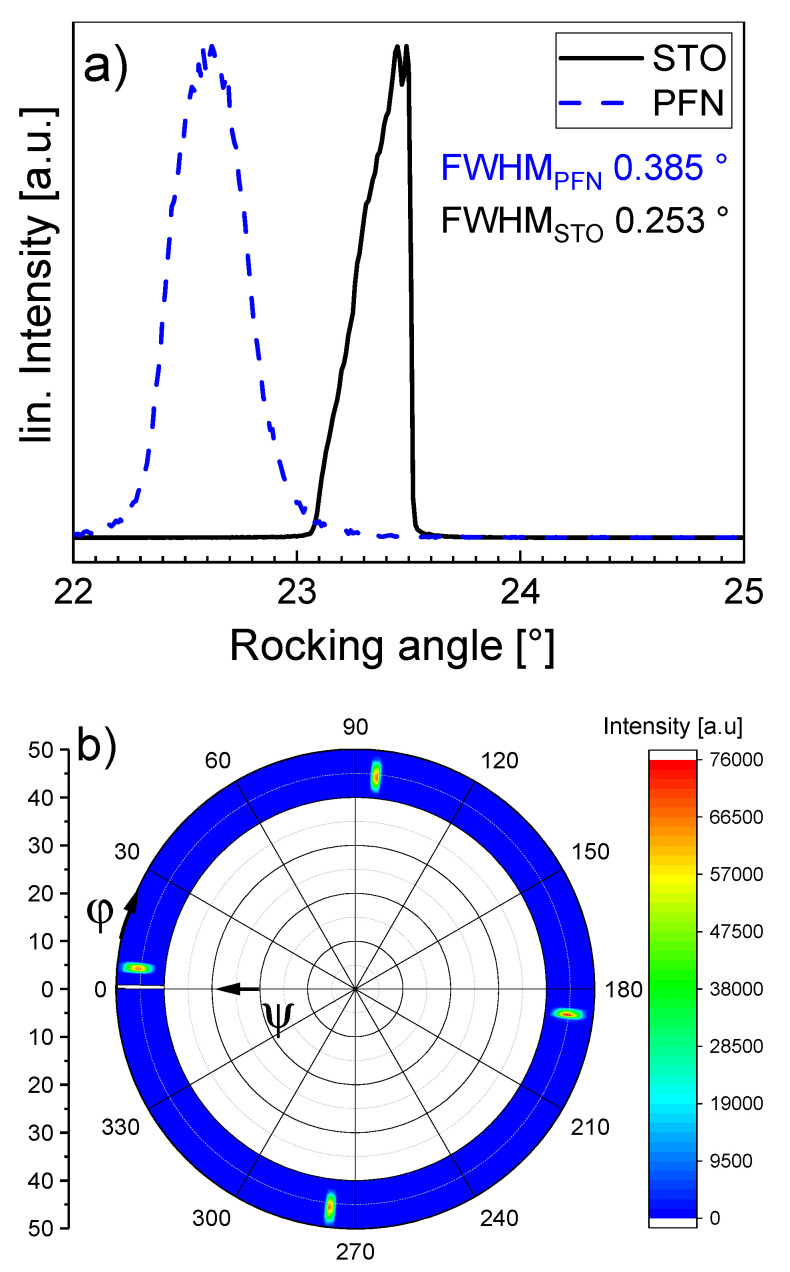
Rocking curve (**a**) and pole figure (**b**) measured for the PFN film deposited on an STO (001) substrate.

In Figure 2, the *θ*-2*θ* XRD pattern for a PFN film deposited on an (001)-oriented STO substrate is shown. The STO and PFN peaks are clearly visible, and no secondary phase is observed, indicating the formation of a phase-pure perovskite layer. The detected PFN peaks correspond to (001) planes of the perovskites structure, which indicates that the film grows epitaxially on the STO (001) substrate. In order to check the crystallinity of the film, a rocking curve was measured (Figure 3a). Here, sharper peaks of the rocking curve indicate a higher degree of crystallinity of the film. The PFN peak is not particularly sharp; the full-width at half maximum (FWHM) is FWHM_PFN_ = 0.385°. However, it should be considered that the rocking curve of the crystalline substrate is also relatively broad, with FWHM_STO_ = 0.253°. As the PFN peak is only 1.5 times broader, we assume the crystallinity of the thin film to be high. For confirmation, we performed additional pole figure measurements, with the result being shown in Figure 3b. The sample only yields four peaks in the *ψ* range from 40° to 50°, which means that it has the same in-plane orientation as the substrate and there is no rotation of individual grains in the layer. Since four peaks are visible, the PFN film must have a fourfold rotation symmetry, which is either tetragonal, or cubic. Bulk PFN has a monoclinic structure [27]; however, mechanical misfit stress may stabilize another structure in epitaxial thin films. Yan et al. [33] investigated the structure of PFN deposited on cubic STO (001) and found that due to the lattice mismatch, the *a* and *b* parameter of the unit cell are equally compressed, and the *c* parameter is expanded, resulting in a tetragonal structure.

**Figure 4:**
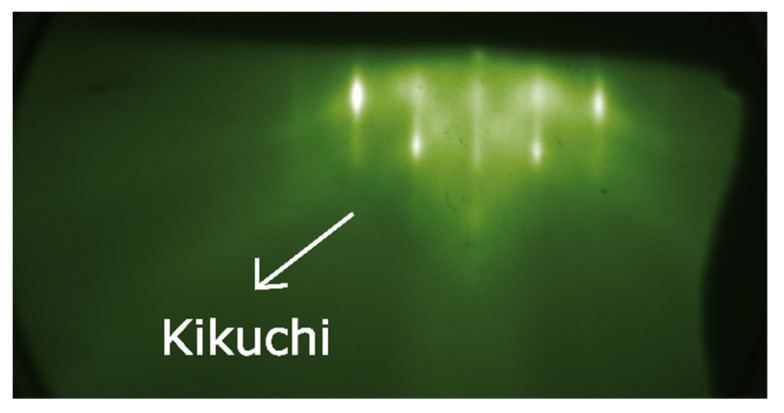
In situ RHEED measurements show the Laue circle and clear Kikuchi lines.

To further investigate the surface quality of the PFN thin film, RHEED measurements were carried out in situ right after deposition (Figure 4). The indication of the Laue circle, the appearance of Kikuchi lines, and an RHEED streaky pattern indicate a smooth surface and an excellent crystalline quality.

In order to estimate the layer thickness, reflectivity measurements were performed. X-ray reflectivity is a technique sensitive to the electronic density (chemical composition), thickness, and roughness and is a reliable method to evaluate the thickness of thin films and multilayers using the following relation:

(1) $$\theta^{2}-\theta_{c}^{2}=m^{2}{\left(\frac{\lambda }{2d}\right)}^{2}$$

where *d* is the thickness, *λ* is the X-ray wavelength, *θ* is the satellite maximum, *m* is the order of reflection, and *θ_c_* is the critical angle of reflectivity. By plotting the maxima *θ^2^* versus the reflection order *m^2^* and performing a linear fit, a value of *d* can be extracted from the slope of the linear fit. This simple method allows determining the thicknesses without simulating the whole intensity profile [34].

**Figure 5:**
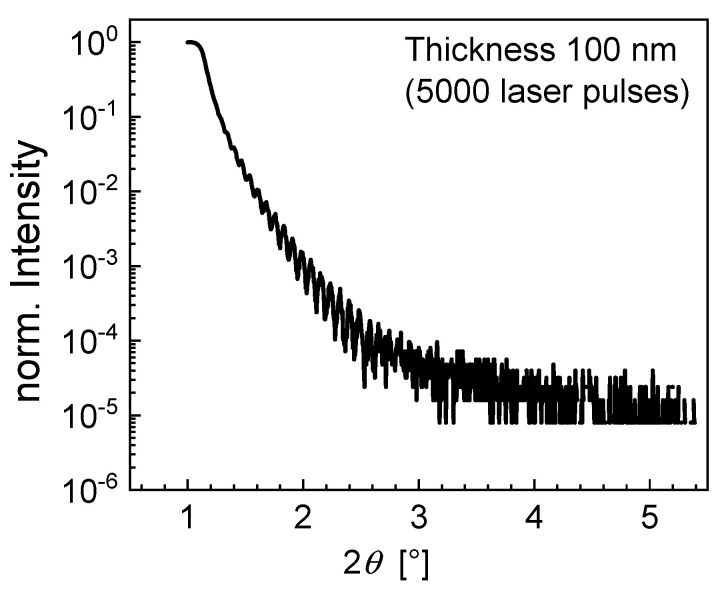
Reflectivity measurement with Kiessig oscillations for a PFN layer deposited with 5000 laser pulses.

Figure 5 shows the reflectivity of the film obtained after 5000 laser pulses, where typical Kiessig oscillations are observed [35]. A large number of oscillations indicates a flat and well-defined film/substrate and film/air interface, which supports the results of the RHEED measurements. By fitting these oscillations using Equation (1), a layer thickness of approximately 100 nm can be estimated. Increasing the layer thickness with a higher deposition time resulted in a drastic increase in the surface roughness.

**Figure 6:**
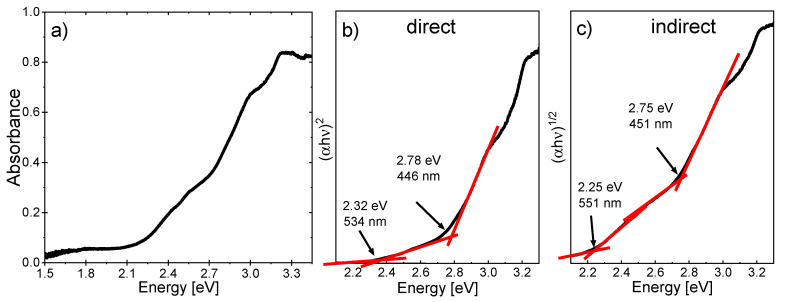
Absorbance spectra for the PFN layer from UV-Vis absorption measurements (**a**). Tauc plots for direct (**b**) and indirect (**c**) band gap.

Current reports about the band gap of PFN in the literature are contradictory. According to the theoretical calculations of Bharti et al. [31], PFN has a direct band gap of 0.43 eV. However, from experimental UV-Vis spectroscopy measurements performed on PFN powders [30], values of the band gap larger than 2.3 eV were obtained. In order to evaluate the band gap of a material, epitaxial films are convenient objects of study. Since the layers are grown epitaxially and are highly oriented, one can expect that the number of defects and related trap states is low. The widely used method for the estimation of the band gap is based on measuring the absorption spectra using UV-Vis spectroscopy. Several absorption edges corresponding to optical transitions can be observed between ~2.3 eV and 3.2 eV in Figure 6a. If the films are homogeneous and not strongly confined, Tauc plots can be applied to the optical absorption curves to determine the optical bandgap of the material [36,37]. The functional form of the Tauc plot depends on the direct or indirect nature of the band gap. Because the character of the band gap for PFN is unknown, the Tauc plots for both cases (Figure 6b,c) were evaluated and yielded similar results with optical transitions at 2.32 eV (2.25 eV) and 2.78 eV (2.75 eV) for the direct (indirect) band gap approximations. These values are similar and in rough agreement with the value of 2.55 eV reported for PFN powders prepared by mechanical activation-assisted synthesis [30].

**Figure 7:**
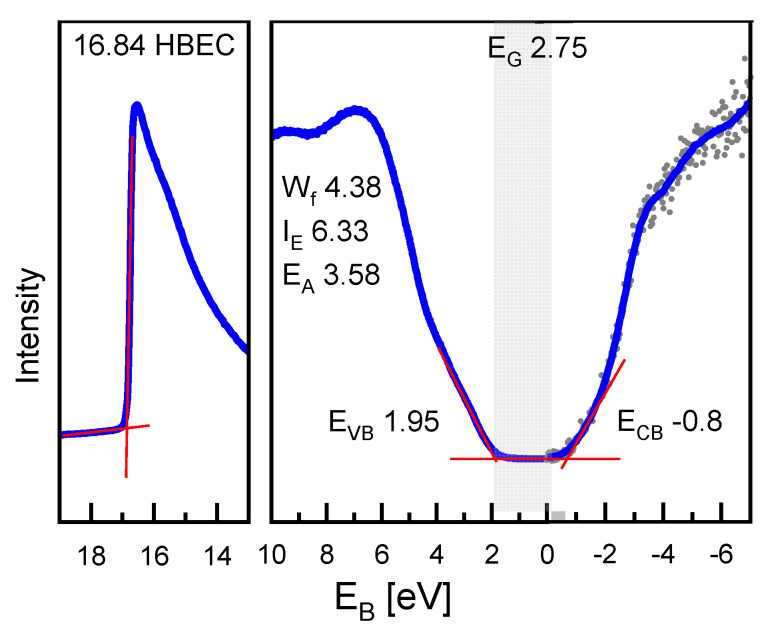
Combined IPES/UPS measurements of the PFN layer deposited on STO/SRO. The band onset, the position of the high binding energy cut-off (HBEC), and extracted energy values are indicated in the graph.

To complement the results of the UV-Vis measurements (Figure 6), additional UPS/IPES measurements (Figure 7) were performed on the PFN layer deposited on STO/SRO. It should be noted that by using such techniques with high surface sensitivity (few nm), surface contamination, e.g., due to the preparation in an oxygen-rich environment, can affect the results. Thus, the calculated values for the work function (*W_f_*), the ionization energy (*I_E_*), and the electron affinity (*E_A_*) may deviate some 100 meV from the actual material values but should be precise enough for the estimation. The obtained results yield a conduction band minimum *E_CB_* at -0.8 eV relative to the Fermi level and the valence band maximum *E_VB_* at 1.95 eV, resulting in the fundamental band gap *E_G, fund_* of 2.75 eV. The fundamental band gap is defined as [38]:

(2) $$E_{G,\;\;fund}=I_{E}-E_{A}=E_{CB}-E_{VB}$$

This value is in good agreement with the high-energy optical transition estimated from the UV-Vis measurements discussed before. While the UPS/IPES measurements give information about the fundamental band gap, UV-Vis absorption measurements detect all kinds of optical transitions, which also includes defect states and band tails. We therefore conclude, from the comparison of the UV-Vis spectroscopy with the UPS/IPES data, that the absorption edge observed at ~2.3 eV (2.25 eV) likely originates from defect levels in the band gap, while the absorption at ~2.78 eV (2.75 eV) corresponds to the fundamental band gap. It was reported in the literature that the band gap value in PZT or PLZT films depends on the film thickness, while no clear trend has been found [39,40,41]. In our case, the band gap for the 100 nm thin films is comparable with the data for the bulk PFN, where the value of 2.67 eV was measured. Since no confinement effects are expected in the 100 nm films, the agreement of the band gaps is another hint towards the great quality of the PFN layer, despite the large surface to volume ratio.

As for the nature of the band gap in PFN, we have to note that most of the literature [12,22,42] considers a direct band gap for perovskite ferroelectrics such as PZT, PLZT, and BFO. As PFN belongs to the same group of materials, the direct band gap is more likely to be correct, although this question cannot be answered conclusively.

**Figure 8:**
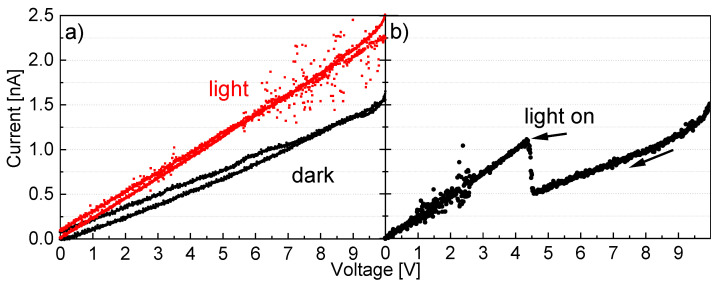
(**a**) TLM response of a sample in dark (black lines) and illuminated (red lines) for a contact distance of 800 µm. (**b**) The sample was illuminated while sweeping.

Notably, the band gap in PFN is much smaller compared to other lead-based perovskite ferroelectrics, which extends the range of absorbed light from ultraviolet to the blue range of the visible spectrum up to around 450 nm. The device efficiency can be expected to be higher than for PZT or PLZT if a photovoltaic effect is also seen in PFN. To make initial tests in this direction, we checked whether the PFN layers react to illumination by generating a photocurrent. For this, a voltage was applied to the structure shown in Figure 1b and swept from −10 V to +10 V. The current flowing through the PFN layer was measured in the dark and under the illumination by a white light source and is displayed in Figure 8. For better visualization, the symmetrical results for both voltage polarities are shown in the positive voltage range. It can clearly be seen that when the sample is illuminated, the current increases. The conductivity rises from 23 S/µm to 35 S/µm, which means that charge carriers are generated by the light (Figure 8a). This increase needs to be confirmed further: When the light was turned on during the voltage sweep at 4.5 V, the sample showed a clear photoconductive response in the form of an instantly increasing current, as seen in Figure 8b.

A relatively low current can be explained by the large distance (800 µm) between the Au electrodes and the strong insulating character of the PFN thin film. However, this might not be the expected photovoltaic effect since the polarization direction is out of plane due to the tetragonal structure, and the electric field is applied in-plane, which makes the electron–hole separation more difficult. Nevertheless, a photoresponse has been proven.

## 4. Conclusions

A pure PFN layer was successfully deposited on a (001)-oriented STO substrate using PLD. It was grown epitaxially with high crystallinity and a tetragonal structure as indicated by the XRD measurements. UV-Vis spectroscopy measurements revealed optical transitions at ~2.32 eV (~2.25 eV) and ~2.78 eV (~2.75 eV) for the direct (indirect) band gap. These results of the UV-Vis spectroscopy are in good agreement with the UPS/IPES measurements indicating a fundamental band gap of ~2.75 eV. The optical transition at lower values is assigned to defects states such as oxygen or lead vacancies. However, the question of direct or indirect band gap could not be answered yet. The band gap value is more promising for photovoltaic applications compared to other lead-based ferroelectrics as PZT, PLZT, or PTO, especially as initial tests under illumination proved the photogeneration of charge carriers in the PFN film.

## Data Availability

The data presented in this study are available on request from the corresponding author.
